# Differential Cadmium Responses in Two *Salvia* Species: Implications for Tolerance and Ecotoxicity

**DOI:** 10.3390/plants15030375

**Published:** 2026-01-25

**Authors:** Douaa Bekkai, Natalizia Miceli, Francesco Cimino, Carmelo Coppolino, Maria Fernanda Taviano, Francesco Cacciola, Giovanni Toscano, Luigi Calabrese, Patrizia Trifilò

**Affiliations:** 1 Department of Chemical, Biological, Pharmaceutical and Environmental Sciences, University of Messina, Viale F. Stagno d’Alcontres, 31, 98166 Messina, Italy; natalizia.miceli@unime.it (N.M.); francesco.cimino@unime.it (F.C.); mariafernanda.taviano@unime.it (M.F.T.); giovanni.toscano@unime.it (G.T.); ptrifilo@unime.it (P.T.); 2 Messina Institute of Technology c/o Department of Chemical, Biological, Pharmaceutical and Environmental Sciences, University of Messina, Viale G. Palatucci 13, 98168 Messina, Italy; carmelo.coppolino@unime.it (C.C.); francesco.cacciola@unime.it (F.C.); 3 Department of Engineering, University of Messina, Contrada di Dio, Sant’Agata, 98158 Messina, Italy; luigi.calabrese@unime.it

**Keywords:** cadmium stress, phenolic metabolism, ecotoxicological assessment, *Salvia officinalis*, *Salvia ceratophylloides*

## Abstract

Heavy metal contamination poses critical challenges for the cultivation of medicinal plants. This study explores cadmium (Cd)-induced morpho-physiological and metabolic responses in *Salvia officinalis* (So) and the rare endemic *Salvia ceratophylloides* (Sc). Plants were exposed to cadmium contamination corresponding to 5 and 10 mg kg^−1^ Cd (100% and 200% of the Italian regulatory limit) and assessed through gas exchange, leaf anatomy, mineral profiling, polyphenol composition, antioxidant activity, and a preliminary ecotoxicological evaluation using the *Artemia salina* lethality bioassay. Cd predominantly accumulated in roots, reflecting a partial exclusion strategy, and caused alterations in leaf traits, water relations, and nutrient balance. While total polyphenols generally declined, species-specific responses emerged: *S. ceratophylloides* increased caffeic acid derivatives, whereas *S. officinalis* accumulated caffeic acid, lithospermic acid A, quercetin 3-O-glucuronide, and apigenin-O-pentoside at the highest Cd exposure. Polyphenol shifts were strongly associated with antioxidant capacity. Despite higher growth sensitivity, *S. ceratophylloides* extracts exhibited no toxicity in the *A. salina* assay, indicating effective metal sequestration and low bioavailability, whereas *S. officinalis* extracts induced moderate to high toxicity. These findings reveal contrasting Cd tolerance and detoxification strategies, highlighting the potential of integrating plant stress physiology with ecotoxicological assessment and phytostabilization approaches to safely cultivate medicinal species on contaminated soils.

## 1. Introduction

Environmental pollution is one of the most critical global challenges, and heavy metals represent a major class of persistent and hazardous contaminants. These elements are released through industrial activities, intensive agriculture, and improper waste disposal, and their accumulation in soil and water severely affects plant physiology. Heavy metals interfere with photosynthesis, disrupt mineral nutrition, and induce oxidative stress, ultimately reducing plant growth and productivity [1]. Among them, cadmium (Cd) is particularly dangerous due to its high mobility, environmental persistence, and strong toxicity even at low concentrations [2,3]. Cd is readily absorbed by plants and disturbs cellular homeostasis by inducing reactive oxygen species (ROS) overproduction, lipid and protein oxidation, DNA damage, and alterations in antioxidant defense pathways [4]. These biochemical disruptions often translate into marked changes in leaf structure, gas-exchange performance, and secondary metabolism. In non-hyperaccumulator species, tolerance to Cd often relies on exclusion and immobilization mechanisms, including preferential root retention and reduced translocation to shoots [5]. These strategies are central to phytostabilization-based approaches, which aim to limit metal mobility and bioavailability while maintaining ecosystem functionality.

Interestingly, heavy metal stress may not only damage plants but can also act as an abiotic elicitor in medicinal and aromatic species, enhancing the biosynthesis of valuable secondary metabolites [6,7]. Several metals, including cadmium, copper and lead, have been reported to increase phenolic antioxidants or modify essential oil composition [8,9,10]. In this context, phenolic compounds play a dual role by contributing to reactive oxygen species scavenging and by chelating metal ions, thereby reducing their cellular toxicity and ecological bioavailability. These responses have important implications for both phytoremediation and the valorization of plant-derived bioactive compounds. However, the magnitude and direction of these effects vary widely according to species, metal type, dose and exposure duration. Depending on these factors, heavy metals may either stimulate or suppress secondary metabolism. For instance, *Ocimum basilicum* showed shifts in essential oil composition under metal exposure, whereas *Mentha spicata* remained unaffected [8].

Within this framework, the genus *Salvia* represents a relevant model because of its high antioxidant properties and diverse secondary metabolite profiles [11,12]. While *Salvia officinalis* L. is widely studied, the rare endemic *Salvia ceratophylloides* Ard. remains largely unexplored. Current research has focused mainly on drought tolerance and biotic interactions [13,14,15], whereas its responses to heavy metal exposure are still unknown. Recent phytochemical data indicates high rosmarinic acid content under non-stressed conditions, suggesting strong antioxidant potential [16].

To address this knowledge gap, this study compares *Salvia ceratophylloides* and *Salvia officinalis* under controlled cadmium exposure. Morphological traits, gas-exchange parameters, phenolic profiles, and in vitro antioxidant capacity (DPPH scavenging, reducing power, and Fe^2+^ chelation) were analyzed, together with a preliminary ecotoxicological assessment of leaf extracts using the *Artemia salina* lethality bioassay. The study aims to elucidate species-specific physiological and metabolic plasticity under Cd stress and to evaluate the ecological and pharmacological implications of metal exposure, contributing to the mechanistic understanding of plant-based mitigation strategies and environmental risk assessment in Cd-contaminated soils.

## 2. Materials and Methods

### 2.1. Plant Material and Growth Conditions

Experiments were carried out using eight-month-old plants of *Salvia ceratophylloides* Ard. (Sc) and *Salvia officinalis* L. (So) (Supplementary Figure S1). Seeds of both species, supplied by the Botanical Garden of the University of Messina, were soaked in distilled water for 24 h and sown in greenhouse trays in October 2023. Approximately one month after germination, seedlings were individually transplanted into 3.5 L plastic pots containing 0.8 Kg of nutrient-enriched potting mix suitable for vegetable cultivation (Comodo, Tercomposti S.P.A., Italy), with pH 7.3 ± 0.1, organic carbon 311 ± 13 g kg^−1^, cation exchange capacity 14.6 ± 3.1 cmol kg^−1^, and electrical conductivity 412 ± 67 µS cm^−1^, as reported by [17].

**Figure 1:**
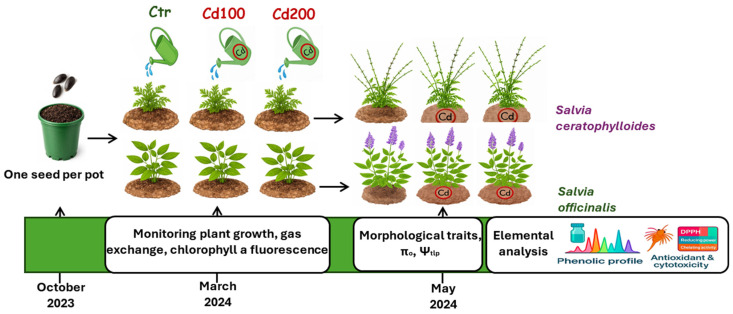
Experimental design. Seeds of *Salvia ceratophylloides* and *Salvia officinalis* were sown in October 2023. Approximately one month after germination, seedlings were individually transplanted into pots and, when they were about 4 months old (March 2024), they were divided into three experimental groups per species. The first group, used as the control, was regularly irrigated with uncontaminated water. The second and third groups were irrigated with water containing cadmium (Cd) to achieve soil contamination levels corresponding to the maximum limit allowed by Italian law for agricultural land (5 mg kg^−1^, hereafter referred to as Cd100) and twice that concentration (Cd200), respectively. From March until the end of the experimental period, gas exchange and chlorophyll *a* fluorescence were measured. In May, before leaf sampling for osmotic potential at full turgor (π_o_), turgor loss point (Ψ_tlp_), mineral content analysis, phenolic profiling, and leaf extract toxicity assays, morphophysiological traits were also measured.

Plant growth took place in the greenhouse facilities of the Department of Chemical, Biological, Pharmaceutical and Environmental Sciences (ChiBioFarAm), University of Messina. The greenhouse was illuminated by LED lamps (Led Spectrum Grow 6, I-GROW s.r.l., Italy), ensuring a 16 h light/8 h dark regime and a photosynthetic photon flux density of 450 µmol m^−2^ s^−1^. Daytime and nighttime temperatures were maintained at approximately 24 °C and 18 °C, respectively. In February 2024, plants were transferred to an open-air terrace at the ChiBioFarAm Department. After one month of acclimation, they were divided into three experimental groups per species (Figure 1). The first group, used as control, was regularly irrigated with uncontaminated water. The second and third groups were irrigated with water containing cadmium (Cd) to achieve soil contamination levels corresponding to the maximum limit allowed by Italian law for agricultural land (5 mg kg^−1^, hereafter referred to as Cd100) and twice that concentration (10 mg kg^−1^, Cd200), respectively (Law No. 46, 22 June 2019).

Specifically, plants assigned to the Cd100 treatment were irrigated with 40 mL of water containing 890 μmol L^−1^ CdCl_2_, corresponding to approximately 5 mg Cd per kg of soil, while plants in the Cd200 treatment received the same volume of water containing 1790 μmol L^−1^ CdCl_2_, equivalent to 10 mg Cd per kg of soil. To ensure homogeneous exposure, the solution was applied slowly and evenly over the surface of each pot, allowing cadmium to infiltrate naturally throughout the soil.

A subset of control plants of *S. officinalis* and *S. ceratophylloides* was previously analyzed to characterize polyphenolic composition and antioxidant capacity. In the present study, these results served as reference for evaluating Cd-induced changes. Data from these control plants are provided in Supplementary Table S4, Supplementary Figures S2 and S3 of this study and in [16].

### 2.2. Morphological Measurements

To evaluate the impact of Cd pollution on plant growth, at the end of the experimental period, once the plants had developing flowers, these samples were utilized to assess plant biomass (*n* = 5). The plants were divided into roots (gently rinsed to remove soil), stems, leaves, and flowers, and subsequently oven-dried at 80 °C for three days to determine their dry weight (DW). This dried material was used to estimate the mineral content of the organs, including Cd content (see below).

Prior to oven-drying, the leaf surface area (A_L_) and thickness (T_L_) of five leaves from different plants and species were measured. A_L_ was determined by scanning leaf images using an HP Scanjet G4050 scanner (USA) and analyzing them using Fiji (Fiji Is Just ImageJ), an open-source platform for biological-image analysis based on ImageJ. T_L_ was calculated as the average measurements taken at the base, middle, and tip of the leaf using a digital caliper with an accuracy of ± 0.01 mm. Leaf mass per area (LMA) was calculated as DW/A_L_. Leaf density was estimated using the ratio LMA/T_L_, while leaf volume was derived from the product T_L_ × LMA.

### 2.3. Gas Exchange, Chlorophyll a Fluorescence and Water Relations Measurements

During the experimental period, the Ciras 4 Portable Photosynthesis System (Amesbury, MA, USA), set at CO_2_ = 400 μmol m^−2^s^−1^, was used to assess the stomatal conductance to water vapor (g_L_), transpiration rate (E_L_), and photosynthetic rate (An) twice a week in at least five leaves from different plants per treatment. Measurements of chlorophyll *a* fluorescence were performed using a portable fluorometer (Handy PEA+, Hansatech, Norfolk, UK) and Fv/Fm was measured as a proxy for the quantum yield of PSII of dark-adapted leaves [18].

At the end of the experimental period, the osmotic potential at full turgor (π_0_) and the leaf turgor loss point (Ψ_tlp_) were determined using a dewpoint hygrometer (Model WP4, Decagon Devices Inc., Pullman, WA, USA) on five plants per species and treatment. Leaves were collected in the early morning (7:00 a.m.) from well-watered plants and their petioles were immersed in distilled water for approximately 1 h to ensure full hydration. Leaves were then sealed in cling film, immersed in liquid nitrogen for 2 min, ground, and stored in sealed plastic containers at −20 °C. Prior to measurement, samples were thawed at room temperature, and the osmotic potential of the expressed sap (π_0__osm) was measured with the dewpoint hygrometer.

The osmotic potential at full turgor (π_0_) and the leaf water potential at the turgor loss point (Ψ_tlp_) were calculated according to [19] using the following equations:

π_0__fit = 0.506 πo_osm − 0.002*LDMC

Ψ_tlp_ = 1.313π0_fit − 0.032

where LDMC (mg g^−1^) is the leaf dry matter content measured on five leaves different from those used for osmotic potential determination but collected from the same plants. Leaves used for LDMC determination were rehydrated for 1 h, and their turgid weight (TW) was measured using an analytical balance. Samples were then oven-dried at 80 °C for 72 h to obtain the dry weight (DW), and LDMC was calculated as: DW/TW.

### 2.4. ICP-OES Mineral Element Analysis

Stem, leaf, root, and flower samples previously used for biomass measurements were also analyzed for cadmium and macro- and micronutrient content. Approximately 2 g of each sample was placed in an acid-prewashed digestion vessel, to which 7 mL of 65% HNO_3_ was added. Samples were introduced into a microwave digestion system (Ethos 1; Milestone, Bergamo, Italy) and treated with a warm-up program of 20 min at 1000 W of microwave power followed by the addition of 1 mL of 30% H_2_O_2_ and further digested for 20 min at 1000 W. After digestion and cooling processes, samples were diluted to a final volume of 25 mL with ultrapure water.

Mineral content was measured using an Avio200 ICP-OES instrument (PerkinElmer, Waltham, MA, USA) equipped with a vertical DualView optical system and an S10 autosampler (PerkinElmer, Waltham, MA, USA). Supplementary Table S1 lists the recommended analytical wavelengths for each element, and the argon line at 420.069 nm was used as an internal standard. Operational parameters for ICP-OES are reported in Supplementary Table S2. Data acquisition and processing were performed using PerkinElmer Syngistix™ for ICP software (version 5.1 PerkinElmer, Waltham, MA, USA). Torch positioning was optimized prior to analysis using the optical optimization routine with the Mn analytical line.

Quantification was performed against external calibration curves prepared from a Certified Reference Material multielement standard solution (ISO 17034, CPAchem, Bogomilovo, Bulgaria). Ultrapure water (1.8 MΩ·cm, Milli-Q, Merck Millipore, Darmstadt, Germany) was used for solution preparation and sample dilution. Calibration curves for all the elements were generated using both calibration and reagent blanks, with correlation coefficients (r^2^) exceeding 0.999. Detection limits (DLs) were determined by analyzing matrix blanks containing the same reagents and volumes as the samples and calculated as DL = 3.3 × σ/S, where σ is the standard deviation of the response and S is the slope of the calibration curve.

### 2.5. Leaf Extraction Procedure

Leaves of *S. officinalis* and *S. ceratophylloides* (control, Cd100, and Cd200) were collected in May 2024. After harvesting, the plant material was lyophilized and finely ground. The powdered leaves were first subjected to a preliminary maceration at 25 °C in 70% ethanol (1:10 *w*/*v*) for 1 h. Extraction was then carried out using 70% ethanol (1:10 *w*/*v*) in an ultrasonic bath at 50 °C for 15 min, and the procedure was repeated twice. The combined filtrates were subsequently evaporated to dryness using a rotary evaporator.

The extraction yields of *S. ceratophylloides* (Sc_Ctr, Sc_Cd100, Sc_Cd200) and *S. officinalis* (So_Ctr, So_Cd100, So_Cd200) extracts, calculated with respect to 100 g of lyophilized plant material, are reported in Supplementary Table S5.

### 2.6. Determination of Polyphenolic Compounds by HPLC-PDA/ESI-MS

Each dried extract was re-dissolved in 70% ethanol and filtered through a 0.45 μm Acrodisc nylon membrane (Merck Life Science, Merck KGaA, Darmstadt, Germany) prior to chromatographic analysis.

LC–MS grade water (H_2_O), acetonitrile (ACN), formic acid, and the polyphenolic standards caffeic acid, apigenin, salvianolic acid B, luteolin-7-O-glucoside, and quercetrin were purchased from Merck Life Science (Merck KGaA, Darmstadt, Germany). For each compound, whose reference material was not available, a semi-quantitative approach was performed, considering the chemical structure and comparable absorptivity coefficients.

Polyphenolic profiles of samples were determined using a Shimadzu HPLC system (Kyoto, Japan) including a CBM-20A controller, two LC-30AD dual-plunger parallel-flow pumps, a DGU-20A3R degasser, a CTO-20AC column oven, a SIL-30AC autosampler, an SPD-M30A photodiode array detector, and an LCMS-2020 mass spectrometer fitted with an electrospray ionization (ESI) source operating in negative ion mode (Shimadzu Kyoto, Japan).

Separations were performed on an Ascentis Express C18 column (150 × 2.1 mm, 2.7 μm; Merck Life Science, Merck KGaA, Darmstadt, Germany). The mobile phase consisted of water (solvent A) and acetonitrile (solvent B), both containing 0.1% formic acid (*v*/*v*). The gradient program was as follows: 0 min—0% B, 10 min—10% B, 20 min—11% B, 30 min—15% B, 50 min—18% B, and 65 min—23% B. The flow rate was set at 0.5 mL min^−1^, and the injection volume was 2 μL. PDA detection was carried out in the range of 190–450 nm, with chromatograms monitored at 330 nm (sampling frequency: 40 Hz; time constant: 0.050 s).

Mass spectrometry parameters were as follows: scan range, *m*/*z* 100–1200; scan speed, 7500 amu s^−1^; event time, 0.3 s; nebulizing gas (N_2_) flow, 1.5 L min^−1^; drying gas (N_2_) flow, 15 L min^−1^; interface temperature, 350 °C; heat block, 300 °C; desolvation line, 300 °C; desolvation line voltage, 1 V; and interface voltage, −4.5 kV.

Quantification of polyphenolic compounds in the extracts was performed using calibration curves constructed for the five standard compounds. Data acquisition and processing were performed using Shimadzu LabSolution software (version 5.97).

### 2.7. In Vitro Antioxidant Assays

*DPPH radical scavenging assay*: The free radical scavenging activity of *S. ceratophylloides* (Sc_Ctr, Sc_Cd100, Sc_Cd200) and *S. officinalis* (So_Ctr, So_Cd100, So_Cd200) extracts was evaluated using the DPPH assay [20]. Sample concentrations ranged from 0.0625 to 2 mg mL^−1^, with butylated hydroxytoluene (BHT) serving as the reference standard. Briefly, 0.5 mL of each extract solution was mixed with 3 mL of 0.1 mmol L^−1^ DPPH methanol solution and incubated in the dark at room temperature for 20 min. Absorbance was measured at 517 nm using a UV-1601 spectrophotometer (Shimadzu, Milan, Italy).

Results were expressed as the mean radical scavenging activity (%) ± standard deviation (SD) and the mean 50% inhibitory concentration (IC_50_) ± SD.

*Reducing power assay*: The reducing power of the extracts was determined by the Fe^3+^-Fe^2+^ transformation method [21]. Sample concentrations ranged from 0.0625 to 2 mg mL^−1^, with BHT and ascorbic acid as reference standards. Each assay involved mixing 1 mL of extract with 2.5 mL of 0.2 mmol L^−1^ phosphate buffer (pH 6.6) and 2.5 mL of 1% potassium ferricyanide, followed by incubation at 50 °C for 20 min. After rapid cooling, 2.5 mL of 10% trichloroacetic acid was added and the mixture centrifuged (10 min, 3000 rpm, 4 °C). The supernatant (2.5 mL) was combined with 2.5 mL of distilled water and 0.5 mL of 0.1% ferric chloride and incubated in the dark at room temperature for 10 min. Absorbance was measured at 700 nm. Results are presented as mean absorbance values ± SD and mean ascorbic acid equivalents per milliliter (ASE mL^−1^) ± SD, based on three independent experiments.

*Ferrous ion (Fe^2+^) chelating activity assay*: The Fe^2+^ chelating ability of the extracts was evaluated by spectrophotometric measurement of the Fe^2+^-ferrozine complex [22]. Sample concentrations ranged from 0.0625 to 2 mg mL^−1^, with EDTA as the reference standard. To 1 mL of extract, 0.05 mL of 2 mmol L^−1^ ferrous chloride and 0.5 mL of methanol were added, followed by 0.2 mL of 5 mmol L^−1^ ferrozine. The mixture was shaken vigorously and incubated in the dark at room temperature for 10 min. Absorbance was measured at 562 nm.

Results are reported as the mean inhibition of the ferrozine-Fe^2+^ complex formation (%) ± SD, based on the average of three independent experiments, and as IC_50_ ± SD.

### 2.8. Artemia Salina Lethality Bioassay

The extract toxicity was preliminarily assessed using the brine shrimp (*Artemia salina* Leach) lethality bioassay [23]. Brine shrimp eggs were hatched in artificial seawater (33 g L^−1^ sea salt in deionized water) under a 60 W lamp at 24–26 °C. Twenty-four hours after hatching, groups of ten larvae were exposed for 24 h to varying concentrations of extracts dissolved in DMSO (10–1000 μg mL^−1^) in artificial seawater. Surviving nauplii were counted, and median lethal concentration (LC_50_) values were calculated using GraphPad Prism 10. Each assay was performed in triplicate. Extracts were classified according to Clarkson’s toxicity criterion, considering non-toxic those exhibiting LC_50_ > 1000 μg mL^−1^ [24].

### 2.9. Statistical Analysis

**Figure 2:**
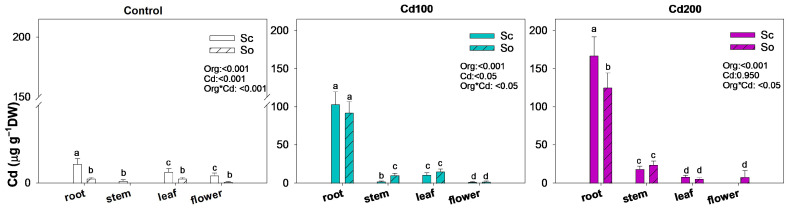
Mean ± SD (*n* = 3) of cadmium concentration in the root, stem, leaf and flower of *Salvia ceratophylloides* (Sc) and *Salvia officinalis* (So) plants grown in good-quality soil (Control) or in the same soil contaminated with two concentrations of cadmium (Cd100 and Cd200). Different letters indicate significant differences between means (*p* < 0.05) based on two-way ANOVA followed by Holm–Sidak post hoc test. Reported *p* values refer to the effects of organ (Org), cadmium concentration (Cd) and their interaction (Sp × Cd). * denotes interaction between factors.

Data was analyzed using the SigmaStat 12.0 statistical package (SPSS Inc., Chicago, IL, USA). A two-way ANOVA followed by a Holm–Sidak post hoc test was used to assess the effects of species (Sc and So) and cadmium treatments (Ctr, Cd100, Cd200) on biomass-related traits, leaf traits and osmotic potential at full turgor and at the turgor loss point (Table 1), total mineral content (Supplementary Table S3) and data of the antioxidant assays (Table 2). Moreover, the same test was performed to assess the effects of organs (root, stem, leaf, flower) and cadmium treatments (Ctr, Cd100, Cd200) on cadmium accumulation (Figure 2) were also evaluated using a two-way ANOVA.

**Figure 3:**
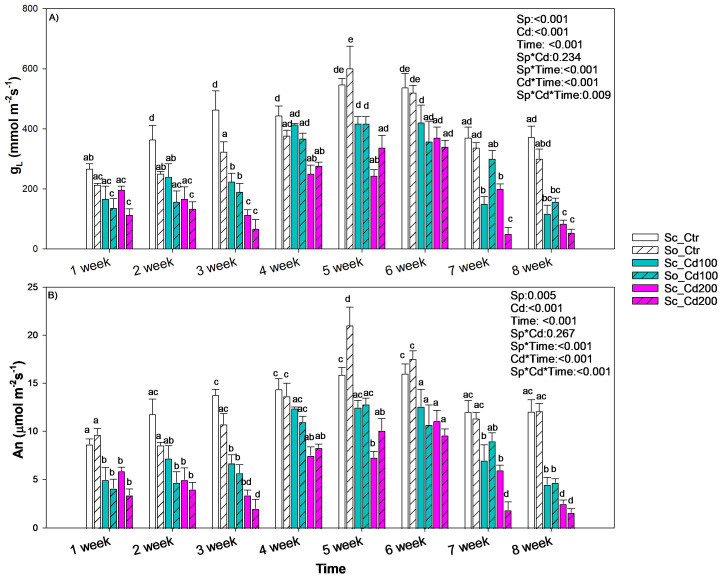
Mean ± SD of (**A**) stomatal conductance to water vapor (g_L_) and (**B**) photosynthesis rate (An) recorded in *Salvia ceratophylloides* (Sc) and *Salvia officinalis* (So) plants grown in good-quality soil (Ctr) or in the same soil contaminated with two concentrations of cadmium (Cd100 and Cd200). Different letters indicate significant differences between means (*p* < 0.05) based on three-way ANOVA followed by Holm–Sidak post hoc test. Reported *p* values refer to the effects of species (Sp), cadmium concentration (Cd), time of exposure (Time) and their interaction (Sp*Cd; Sp*Time; Cd*Time, Sp*Cd*Time). * denotes interaction between factors.

A three-way ANOVA followed by a Holm–Sidak post hoc test was applied to test the effects of species, cadmium treatment, and exposure time on g_L_ and A_n_ (Figure 3).

## 3. Results

In the control plants, cadmium concentrations in all organs were negligible, remaining below 1.5 µg g^−1^ DW (Figure 2). Under cadmium-contaminated soil, the highest accumulation occurred in the roots, reaching approximately 100 µg g^−1^ DW in Cd100 plants and over 120 µg g^−1^ DW in Cd200 plants. At the Cd200 dose, *S. ceratophylloides* accumulated significantly more cadmium in the roots than *S. officinalis* (166.9 ± 24.7 vs. 125.0 ± 19.3 µg g^−1^ DW, respectively). By contrast, leaf cadmium concentrations were relatively similar between species under both treatments, averaging around 10 µg g^−1^ DW (Figure 1 and Table 1).

Interestingly, cadmium was also detected in the flowers of *S. officinalis* under the Cd200 treatment (approximately 8.2 µg g^−1^ DW), while the same exposure inhibited flower production in *S. ceratophylloides*.

Cadmium exposure significantly impaired the growth of *S. ceratophylloides*, particularly under the Cd200 treatment, which resulted in markedly reduced plant height, lower dry weights of leaves, and complete inhibition of flowering compared with both the control and Cd100 plants (Table 1). A different pattern was observed in *S. officinalis*, where cadmium exposure did not significantly alter biomass production.

Leaf traits were strongly influenced by Cd in both species. Cadmium exposure resulted in thicker leaves, but with lower leaf density and reduced leaf mass per area (Table 1). LDMC values also decreased in both species, indicating that leaves became structurally less compact and more succulent under Cd stress. Water-relation traits supported this interpretation: both *S. officinalis* and *S. ceratophylloides* showed increased saturated water content when exposed to cadmium, a response that likely contributed to diluting Cd within leaf tissues (Table 1). Finally, cadmium exposure induced osmotic adjustments that lowered the turgor loss point (Table 1).

The gas exchange of the two species exposed to cadmium showed a similar temporal pattern (Figure 3). After three weeks of exposure, cadmium in the soil caused stomatal closure and a reduction in photosynthetic rate compared with the control, regardless of metal concentration or species. From the fourth to the sixth week, both species exhibited a significant increase in gas exchange under cadmium exposure. In *S. ceratophylloides* values even approached those of the control plants. After the seventh week, however, gas exchange declined again in both species, independently of Cd concentration, with Sc_Cd200 and So_Cd200 showing the lowest values among all treatments (Figure 3). Conversely, Fv/Fm, used as a proxy of photosystem II efficiency, remained unchanged in both species and across all treatments (≈0.8).

**Figure 4:**
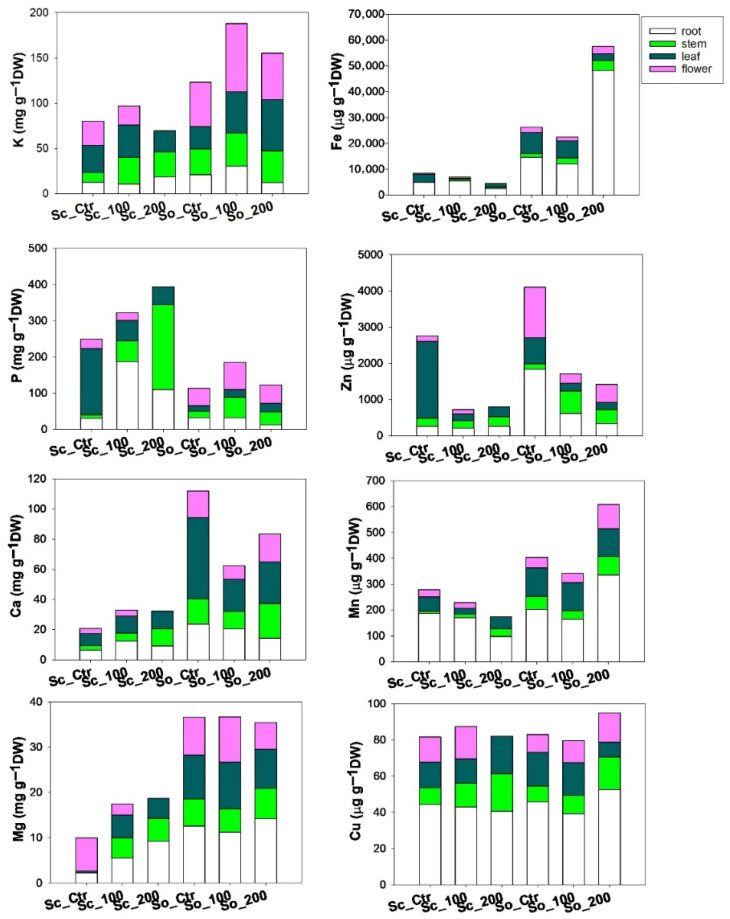
Values of concentration of macronutrients (K, P, Ca, Mg) and micronutrients (Fe, Zn, Mn, Cu) across different plant organs (root, stem, leaf, flower) measured in *Salvia ceratophylloides* (Sc) and *Salvia officinalis* (So) plants grown in good-quality soil (Ctr) or in the same soil contaminated with two concentrations of cadmium (Cd100 and Cd200).

The presence of cadmium in the soil affected not only total nutrient content but also nutrient partitioning among plant organs, with *S. ceratophylloides* (Sc) and *S. officinalis* (So) showing distinct allocation strategies under heavy metal stress (Figure 4, Supplementary Table S3). In Sc, phosphorus increased under cadmium exposure, whereas iron, zinc and manganese decreased, particularly in roots and stems at the higher Cd level. In contrast, So showed an opposite trend: macronutrients P and Ca decreased under cadmium treatment. Among micronutrients, Fe, Mn increased markedly—especially in the roots and under the Cd200 treatment-whereas zinc decreased in response to cadmium.

**Figure 5:**
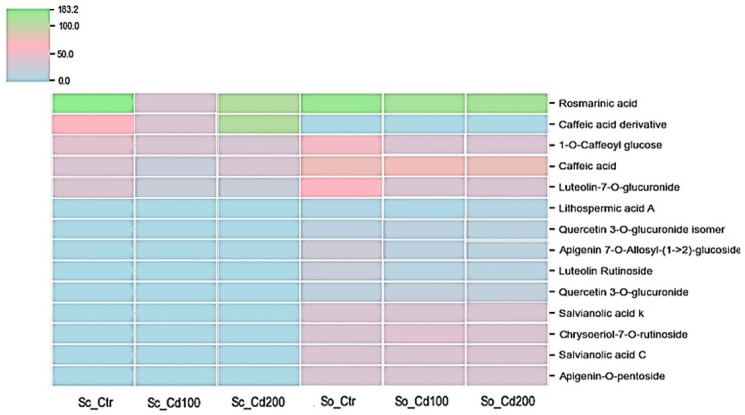
Heat map of phenolic compound variation measured in *Salvia ceratophylloides* (Sc) and *Salvia officinalis* (So) plants grown in good-quality soil (Ctr) or in the same soil contaminated with two concentrations of cadmium (Cd100 and Cd200). Concentration increases from blue to green, according to the color scale.

The two *Salvia* species differed markedly in their phenolic profiles (Figure 5, Supplementary Table S4). Cadmium exposure induced distinct, compound-specific changes in the phenolic composition of both *S. officinalis* and *S. ceratophylloides*, compared with the values reported in plants grown in good quality soil by [16]. Sc showed a concentration-dependent biphasic response in several phenolic compounds. Under Cd100, all five identified metabolites, including rosmarinic acid, caffeic acid derivatives, 1-O-caffeoyl glucose, luteolin-7-O-glucuronide and quercetin glucuronides, showed a marked decline. However, at the higher cadmium concentration (Cd200), caffeic acid and caffeic acid derivative increased significantly relative to the control. Moreover, rosmarinic acid also increased in this species under Cd200 compared with Cd100 (Figure 5, Supplementary Table S4).

In *S. officinalis*, cadmium treatment caused pronounced reductions in most of the 13 quantified metabolites, with decreases ranging from approximately 20% to 70% relative to the control (Supplementary Table S4, Figure 5). The largest declines were observed for luteolin rutinoside and quercetin-3-O-glucuronide isomer. However, some metabolites, including caffeic acid, lithospermic acid, quercetin-3-O-glucuronide and apigenin-O-pentoside accumulated to higher levels under cadmium stress, particularly at Cd200. Notably, lithospermic acid A decreased to 73% of the control value under Cd100 but doubled in concentration under Cd200.

The radical scavenging capacity of the extracts was evaluated by the DPPH assay (Supplementary Figure S2). According to [16], the control extracts (So_Ctr and Sc_Ctr) showed strong antioxidant activity. All the *S. ceratophylloides* extracts retained measurable radical scavenging activity, though with distinct differences across treatments. Sc_Ctr demonstrated the strongest activity, achieving ~86% inhibition already at 0.5 mg mL^−1^, with an IC_50_ of 0.174 ± 0.007 mg mL^−1^ (Table 2). By comparison, Cd exposure caused a marked decline in activity: Sc_Cd200 maintained moderate performance (IC_50_ = 0.345 ± 0.019 mg mL^−1^), while Sc_Cd100 was the most affected, with the highest IC_50_ (0.652 ± 0.035 mg mL^−1^). For *S. officinalis* extracts, both So_Ctr and So_Cd100 exhibited comparable radical scavenging activity, reaching approximately 90% inhibition at the highest tested concentrations (0.5–2 mg mL^−1^), a performance like the synthetic antioxidant BHT (Supplementary Figure S1). This trend is also reflected in their IC_50_ values, which were nearly identical (0.225 ± 0.007 and 0.275 ± 0.005 mg mL^−1^, respectively) (Table 2). In contrast, So_Cd200 showed reduced efficacy, with a higher IC_50_ value (0.376 ± 0.024 mg mL^−1^), indicating that cadmium stress at this concentration impaired the antioxidant potential.

The reducing power capacity of the extracts was evaluated through the Fe^3+^-Fe^2+^ transformation assay (Supplementary Figure S3). Both species showed mild to moderate reducing activity, which increased with concentration but remained consistently lower than the BHT standard. In *S. officinalis*, cadmium exposure resulted in a clear reduction in activity, with So_Cd100 and So_Cd200 performing below the control extract across the tested range. Similarly, in *S. ceratophylloides*, activity was attenuated under stress, with Sc_Cd100 showing the weakest response, followed by Sc_Cd200. When comparing the two species, *S. ceratophylloides* demonstrated higher reducing power overall, although its sensitivity to cadmium stress was more pronounced than that of *S. officinalis*. These findings highlight a common trend of stress-induced reduction in ferric reducing capacity, with species-specific differences in the magnitude of decline.

The ferrous ion chelating activity of the extracts is shown in Supplementary Figure S4. In *S. ceratophylloides* growing into soil with cadmium (Sc_Cd100, Sc_Cd200), chelating activity was consistently higher respect to the plants growing in good quality soil. Extracts from heavy metal stressed leaves outperformed the control at most concentrations, with IC_50_ values of 0.371 ± 0.003 mg mL^−1^ (Sc_Cd100) and 0.205 ± 0.004 mg mL^−1^ (Sc_Cd200), compared to 0.471 ± 0.011 mg mL^−1^ for the control (Table 2). Only at the highest concentration (2 mg mL^−1^) the activities converge. Conversely, *S. officinalis* growing under cadmium pollution (So_Cd100, So_Cd200), a weak ferrous ion chelating activity of about 28% occurred only at 2 mg mL^−1^. IC_50_ values above 2 mg mL^−1^ confirmed the limited chelating capacity of this species, further reduced under cadmium stress.

**Figure 6:**
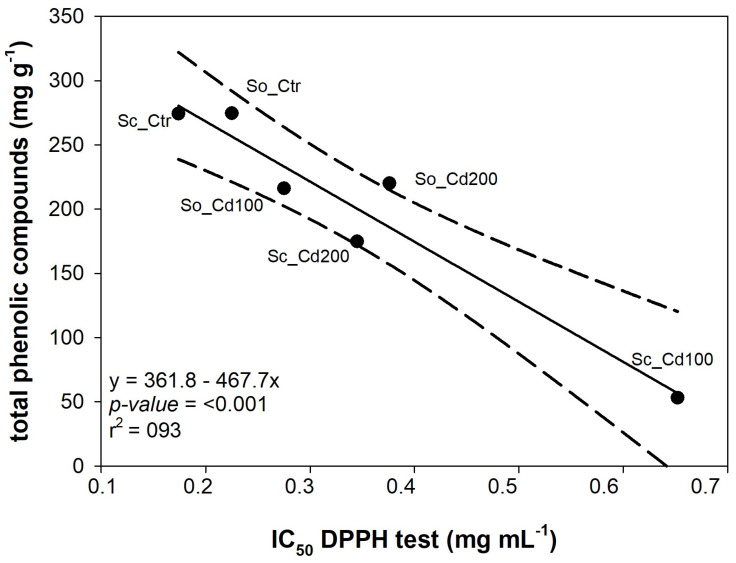
Relationship between the total amount of phenolic compounds detected by HPLC-PDA/ESI-MS analysis and the concentration of extract required to scavenge 50% of DPPH free radicals (IC_50_ DPPH test) measured in *Salvia ceratophylloides* (Sc) and *Salvia officinalis* (So) grown under control (Ctr) or cadmium-contaminated soil (Cd100, Cd200). Linear regression and 95% confidence interval are shown by the solid line and dotted lines, respectively. The equations of the fitted linear regression and the corresponding *p*-value and R^2^; value are reported.

Interestingly, free radical scavenging activity (measured as IC_50_ in the DPPH assay) was tightly correlated with the total phenolic content in the leaves (Figure 6).

The toxicity of the extracts was assessed by the *Artemia salina* leach bioassay. The obtained results of the bioassay carried out for *S. ceratophylloides* extracts (Sc_Ctr, Sc_Cd100 and Sc_Cd200) showed the absence of toxicity against brine shrimp larvae for all the extracts based on the Clarkson’s toxicity criterion applied for the assessment of the degree of toxicity (LC_50_ > 1000 μg mL^−1^) after 24 h of exposure to the extracts (Table 3).

On the other hand, *S. officinalis* extracts (So_Ctr, So_Cd100 and So_Cd200) showed toxicity in the following order: So_Ctr > So_Cd200 > So_Cd100, with LC_50_ values equal to 79.27 ± 11.61, 92.99 ± 9.91 and 346.5 ± 0 μg mL^−1^, respectively (Table 3).

## 4. Discussion

### 4.1. Morpho-Physiological Traits

The two *Salvia* species showed distinct morpho-physiological responses to Cd exposure. In both species, Cd accumulated predominantly in the roots, with limited translocation to aerial organs, a pattern typical of non-hyperaccumulator species and generally interpreted as a protective mechanism that restricts Cd movement toward photosynthetic and reproductive tissues [25,26,27]. *S. ceratophylloides* accumulated significantly higher Cd concentrations in the roots than *S. officinalis*, suggesting greater extraction capacity but also higher sensitivity to metal stress. This susceptibility manifested as substantial growth inhibition and complete suppression of flowering at the highest Cd concentration. In contrast, *S. officinalis*-maintained biomass across treatments, indicating a more efficient detoxification or compartmentalization strategy that mitigates Cd interference with key metabolic processes. Despite the reduction in growth and photosynthetic performance observed under Cd exposure, chlorophyll fluorescence parameters remained unaffected in both species, indicating that the photochemical efficiency of PSII was preserved at the applied Cd levels. The decline in net photosynthetic rate was therefore not associated with photochemical dysfunction but was accompanied by a reduction in CO_2_ assimilation, suggesting that photosynthesis was mainly limited by restricted CO_2_ diffusion rather than by damage to the photosynthetic apparatus. This pattern is consistent with a predominantly stomatal limitation of photosynthesis under moderate metal stress, where reduced CO_2_ availability constrains carbon fixation while photochemical processes remain functional.

Notably, despite reduced growth, *S. ceratophylloides* exhibited no mortality even at the highest Cd level and showed increases in some phenolic compounds (see below comments), indicating that biochemical plasticity may partially compensate for morphological impairment. From an ecotoxicological perspective, the strong root sequestration of Cd observed in both species reduces the immediate risk of metal transfer to aerial biomass and higher trophic levels; however, the contrasting growth sensitivity and metabolic responses highlight species-specific differences in stress cost and potential environmental impact.

Cd exposure produced significant structural and functional alterations in leaves. Both species developed thicker but less dense leaves with reduced leaf dry matter content (LDMC), suggesting increased succulence, a common response in plants exposed to heavy metal stress, also observed in tomato and other species [18,28]. Increased leaf thickness and succulence enlarge cellular volume, reducing the local concentration of Cd and helping shield sensitive organelles from damage. These anatomical changes were associated with modifications in water relations: both species showed higher saturated water content. Nevertheless, more negative osmotic potential and turgor loss point values were recorded under Cd stress, indicating enhanced osmotic adjustment likely driven by the accumulation of compatible solutes and/or metal-chelating molecules [29]. While increased succulence may reduce intracellular Cd concentration, this structural adjustment alone does not necessarily translate into reduced ecotoxicological risk, which depends on metal speciation, compartmentalization, and the chemical nature of plant secondary metabolites. Notably, under heavy metal stress, some species increase leaf succulence, which can help dilute the concentration of potentially toxic ions in the tissues, without activating active osmotic regulation [18,28]. In contrast, more tolerant species actively accumulate compatible solutes such as proline, soluble sugars, and phenolic compounds, thereby lowering Ψ_tlp_ while maintaining cellular function under metal stress [30,31]. This response allows tolerant species to cope with higher metal concentrations by combining structural (succulence) and biochemical (solute accumulation) strategies, whereas species less able to accumulate solutes rely predominantly on water uptake and tissue swelling.

Interestingly, total iron concentration decreased in Sc but increased in Cd-treated So plants. This result may initially appear inconsistent with literature reports describing Cd-induced iron deficiency [32,33]. However, these studies mainly refer to Fe deficiency in leaves. In line with this, in our study leaf Fe concentrations were indeed lower in Cd-exposed plants of both species compared to the controls. Similar responses have been reported in several plant species, in which Cd exposure reduces Fe translocation to the shoots while promoting Fe retention in the roots [34,35,36]. The enhanced Fe retention observed in roots under Cd exposure suggests an active restriction of long-distance Fe transport. Cadmium is known to impair Fe loading into the xylem, thereby limiting its translocation to aerial tissues [5,37,38]. In addition, soil properties and interactions among micronutrients may have contributed to the increased Fe concentrations detected in roots. By immobilizing Fe within root tissues, plants may limit Cd co-translocation to the shoots; however, this protective strategy inevitably disrupts Fe homeostasis in aerial organs, highlighting the close physiological interplay between Cd detoxification mechanisms and Fe nutrition. Moreover, Cd exposure can modify rhizosphere chemistry, increasing the mobility and availability of Fe as well as other micronutrients such as Mn, Cu and Ni [39], which may further contribute to Fe accumulation in root tissues. Thus, Fe retention in roots under Cd exposure represents a protective mechanism against metal translocation but simultaneously induces secondary nutritional stress in shoots, which may indirectly influence plant fitness and the ecological performance of Cd-exposed populations.

Overall, although Cd exposure induces substantial morpho-physiological alterations, particularly severe in *S. ceratophylloides*, both species activate compensatory biochemical mechanisms. These processes, explored in the next section, help explain the survival of both *Salvia* species under high Cd loads and are relevant to assessing their ecological resilience and potential applications.

### 4.2. Phenolic Metabolism and Antioxidant Responses

*S. ceratophylloides* showed a biphasic, hormetic response to cadmium. Moderate Cd exposure (Cd100) caused a decline in most phenolics and a temporary reduction in radical scavenging, reflecting initial stress. At higher Cd (Cd200), specific metabolites, particularly caffeic acid derivative, increased, restoring antioxidant activity and enhancing Fe^2+^ chelation, while reducing power remained moderate. These results indicate an inducible phenolic response that effectively counteracts Cd-induced oxidative pressure. In this context, the recovery of antioxidant capacity at higher Cd levels likely contributed to maintaining redox homeostasis and preventing excessive ROS accumulation at the chloroplast level, thereby preserving the functional integrity of the photosynthetic apparatus, as reflected by the stability of chlorophyll fluorescence parameters observed under Cd exposure.

These findings highlight that phenolic induction under metal stress can represent a double-edged sword, enhancing antioxidant protection while potentially generating compounds with different ecological and toxicological profiles. Toxicity bioassay confirmed that these metabolic adjustments did not produce harmful compounds, highlighting functional resilience despite stress. In contrast, *S. officinalis* showed a more conservative response: several phenolics decreased, but key metabolites such as rosmarinic acid, lithospermic acid A, apigenin-O-pentoside, and quercetin-3-O-glucuronide remained relatively abundant, preserving moderate antioxidant capacity. Radical scavenging declined slightly, and Fe^2+^ chelation remained limited, indicating less inducible plasticity. These results are consistent with previous studies in *Salvia sclarea* and other species, where Cd exposure increased non-enzymatic antioxidants, including total phenolics, anthocyanins, and carotenoids, enhancing ROS detoxification and stress tolerance [40,41,42,43]. Phenolic upregulation is linked to the phenylpropanoid pathway and increased PAL activity, often supporting enzymatic antioxidant systems [44,45]. High phenolic and anthocyanin production under metal stress is also considered advantageous for phytoremediation and phytostabilization [46].

The *A. salina* bioassay highlighted ecotoxicological relevance: although the *A. salina* assay represents a simplified screening tool, it provides an integrative indication of the biological effects of complex plant extracts, complementing chemical and physiological analyses. *S. ceratophylloides* maintained antioxidant defenses without producing toxic metabolites, indicating environmental safety, whereas *S. officinalis* generated moderately toxic compounds despite preserving antioxidants.

In summary, *S. ceratophylloides* relieve dynamic, inducible metabolic adjustments, conferring Cd tolerance and low ecotoxicological risk, making it a promising candidate for phytoremediation and safe cultivation on contaminated soils. *S. officinalis* employs a less flexible strategy, preserving essential defenses but with potential environmental implications. These species-specific responses underline the importance of linking plant stress physiology with ecotoxicological outcomes in evaluating plants for environmental remediation [40,41,42,43].

Collectively, these results show that metal-induced shifts in phenolic metabolism underline both the resilience of *Salvia* species to Cd stress and the potential ecological impact of their bioactive compounds.

## 5. Conclusions

*Salvia ceratophylloides* displays a dynamic and inducible phenolic response under cadmium stress, enhancing antioxidant capacity and metal-chelating potential while remaining non-toxic to Artemia salina larvae. These traits support its suitability for phytostabilization and for the environmentally safe cultivation of medicinal plants on Cd-contaminated soils. By contrast, *S. officinalis* adopts a more conservative metabolic strategy, maintaining key antioxidant compounds; however, control leaf extracts exhibited the highest ecotoxicity among treatments. This suggests that the production of potentially ecotoxic metabolites may represent a species-specific trait rather than a Cd-induced response. Overall, these findings highlight the importance of integrating plant stress physiology with ecotoxicological assessment when evaluating the environmental safety of medicinal plant cultivation.

## Figures and Tables

**Table 1:** Mean ± SD of morpho-physiological traits measured *Salvia ceratophylloides* (Sc) and *Salvia officinalis* (So) plants grown in good-quality soil (Ctr) or in the same soil contaminated with two concentrations of cadmium (Cd100 and Cd200). Different letters indicate significant differences between means (*p* < 0.05) based on two-way ANOVA followed by Holm–Sidak post hoc test. Reported *p* values refer to the effects of species (Sp), cadmium concentration (Cd) and their interaction (Sp*Cd). For plant height (H_plant_) and dry weight parameters (DW_flower_, DW_leaf_, DW_stem_, DW_root_), values are based on five biological replicates (*n* = 5 plants, one plant per pot). For leaf functional and water relation traits (T_L_: leaf thickness; LMA: leaf mass area; LDMC: leaf dry matter content; SWC: saturated water content; π_o_: osmotic potential at full turgor; Ψ_tlp_: leaf water potential at turgor loss point. values are based on five leaves collected from different plants (*n* = 5). * denotes interaction between factors.

	Sc_Ctr	Sc_Cd100	Sc_Cd200	So_Ctr	So_Cd100	So_Cd200	Sp	Cd	Sp*Cd
**H_plant_ (cm)**	66.2 ± 2.8 a	63.0 ±14.8 a	33.3 ± 4.8 b	43.0 ±4.2 c	36.7 ± 0.5 bc	47.0 ± 2.2 c	0.048	0.009	<0.001
**DW_flower_ (g)**	0.4 ± 0.1	0.6 ± 0.6	0	0.6 ± 0.1	0.72 ± 0.3	1.9 ± 1.2	0.044	0.554	0.073
**DW_leaf_ (g)**	3.2 ± 0.8 a	4.6 ± 0.1 a	1.6 ± 0.3 b	4.5 ± 0.8 ac	3.8 ± 0.7 ac	5.2 ± 1.4 c	0.011	0.373	0.006
**DW_stem_ (g)**	2.3 ± 0.5	4.0 ± 0.9	2.0 ± 0.4	5.9 ± 0.5	5.0 ± 2.3	3.3 ± 0.5	0.009	0.074	0.222
**DW_root_ (g)**	1.4 ± 0.2	2.0 ± 1.1	1.4 ± 01	1.8 ± 1.0	2.1 ± 1.1	1.9 ± 0.3	0.465	0.683	0.960
**T_L_ (mm)**	0.33 ± 0.01 a	0.39 ± 0.01 a	0.49 ±0.02 b	0.27 ± 0.01 c	0.42 ± 0.01 a	0.69 ± 0.10 ac	0.996	<0.001	0.026
**Leaf density (g cm^−3^)**	0.19 ± 0.03 a	0.10 ± 0.01 b	0.07 ± 0.01 c	0.33 ± 0.08 d	0.16 ± 0.03 a	0.06 ± 0.01 c	<0.001	<0.001	0.002
**LMA (g m^−2^)**	64.5 ± 9.6	39.8 ± 2.9	32.5 ± 3.8	89.4 ± 20.8	67.5 ± 11.5	41.9 ± 6.1	<0.001	<0.001	0.24
**LDMC (mg g^−1^)**	0.16 ± 0.02 a	0.11 ± 0.01 b	0.09 ±0.00 b	0.23 ± 0.03 c	0.21 ±0.03 c	0.15 ± 0.01 a	<0.001	<0.001	0.003
**SWC (g g^−1^)**	5.54 ± 0.84 a	8.45 ± 0.19 b	9.04 ± 0.27 b	3.41 ± 0.53 c	3.94 ± 0.83 c	5.51 ± 0.31 a	<0.001	<0.001	<0.001
**π_o_ (MPa)**	−0.67 ± 0.04	−0.82 ± 0.08	−0.81 ± 0.03	−0.99 ± 0.04	−1.12± 0.03	−1.14 ± 0.04	<0.001	<0.001	0.753
**Ψ_tlp_ (MPa)**	−0.91 ± 0.05	−1.11 ± 0.1	−1.08 ± 0.03	−1.33 ± 0.06	−1.50 ± 0.03	−1.53 ± 0.05	<0.001	<0.001	0.596

**Table 2:** Mean 50% inhibitory concentration (IC_50_) by DPPH free radical scavenging activity and by Fe^2+^-chelating activity assays, and ascorbic acid equivalents (ASE mL^−1^) by reducing power assay of leaf extracts from *Salvia ceratophylloides* (Sc) and *Salvia officinalis* (So) grown under control (Ctr) or cadmium-contaminated soil (Cd100, Cd200). Data are expressed as mean ± SD (*n* = 3). Standards: BHT for DPPH and reducing power; EDTA for Fe^2+^ chelation. Different letters within the same column indicate significant differences between mean values (two ANOVA followed by Tukey’s multiple comparisons test, *p* < 0.05). * denotes interaction between factors.

	IC_50_, DPPH (mg mL^−1^)	Reducing Power (ASE mL^−1^)	IC_50_, Fe^2+^ Chelating Activity (mg mL^−1^)
Sc_Ctr	0.174 ± 0.007 a	10.169 ± 0.508 a	0.471 ± 0.011 a
Sc_Cd100	0.652 ± 0.035 b	24.785 ± 2.866 b	0.371 ± 0.003 b
Sc_Cd200	0.345 ± 0.019 c	4.267 ± 0.146 c	0.205 ± 0.004 c
So_Ctr	0.225 ± 0.007 d	9.174 ± 0.186 a	>2 d
So_Cd100	0.275 ± 0.005 e	25.146 ± 1.585 b	>2 d
So_Cd200	0.376 ± 0.024 c	12.536 ± 0.778 a	>2 d
Standard	BHT 0.070 ± 0.001	BHT 1.443 ± 0.021	EDTA 0.0067 ± 0.0003
Sp	<0.001	0.015	<0.001
Cd	<0.001	<0.001	<0.001
Sp*Cd	<0.001	0.004	<0.001

**Table 3:** *Artemia salina* lethality bioassay (median lethal concentration, LC_50_) of leaf extracts from *Salvia ceratophylloides* (Sc) and *Salvia officinalis* (So) grown under control (Ctr) or cadmium-contaminated soil (Cd100, Cd200). Data are expressed as mean ± SD (*n* = 3).

	LC_50_ (µg mL^−1^)
Sc_Ctr	>1000
Sc_Cd100	>1000
Sc_Cd200	>1000
So_Ctr	79.27 ± 11.61
So_Cd100	346.5 ± 0
So_Cd200	92.99 ± 9.91

## Data Availability

The original contributions presented in the study are included in the article/Supplementary Material, further inquiries can be directed to the corresponding author.

## Supplementary Materials

The following supporting information can be downloaded at https://www.mdpi.com/article/10.3390/plants15030375/s1: Table S1: Analytical lines length (nm) used for analysis. Table S2: Operational conditions for ICP-OES analysis. Table S3: Mean values ± SD (*n* = 3 of macronutrients (K, P, Ca, Mg) and micronutrients (Fe, Zn, Mn, Cu) total concentration measured in *Salvia ceratophylloides* (Sc) and *Salvia officinalis* (So) plants grown in good-quality soil (Ctr) or in the same soil contaminated with two concentrations of cadmium (Cd100 and Cd200). Different letters indicate significant differences between means *p* < 0.05) based on two-way ANOVA followed by Holm–Sidak post hoc test. Reported *p* values refer to the effects of species (Sp), cadmium concentration (Cd) and their interaction (Sp*Cd). Table S4: Concentration of phenolic compounds in leaf extracts of *Salvia ceratophylloides* (Sc) and *Salvia officinalis* (So) grown in soil contaminated with two cadmium concentrations (Cd100 and Cd200). Data are reported as absolute concentration values and as percentage changes relative to the levels observed in plants grown in uncontaminated soil. Table S5: Percentage yields of *S. ceratophylloides* (Sc) and *S. officinalis* (So) leaf extracts. Plants were grown either in good-quality soil (Sc_Ctr and So_Ctr) or in soil contaminated with cadmium at two different concentrations (Sc_Cd100, Sc_Cd200 and So_Cd100, So_Cd200). For further details, see the main text. Figure S1: Photographs of *Salvia ceratophylloides* and *Salvia officinalis* and details of their leaves recorded at the end of the experimental period. C: plants grown in uncontaminated soil. Cd100 and Cd200: plants grown under two cadmium contamination levels (for details, see Section 2.1). Figure S2: Free radical scavenging activity (DPPH test) of hydroalcoholic leaf extracts from *Salvia ceratophylloides* (Sc) and *Salvia officinalis* (So) plants grown in good-quality soil (Ctr) or in the same soil contaminated with two concentrations of cadmium (Cd100 and Cd200). Data are expressed as the mean ± SD of three independent experiments (*n* = 3). Figure S3: Reducing power of hydroalcoholic leaf extracts from *Salvia ceratophylloides* (Sc) and *Salvia officinalis* (So) plants grown in good-quality soil (Ctr) or in the same soil contaminated with two cadmium concentrations (Cd100 and Cd200), evaluated by spectrophotometric detection of Fe^3+^–Fe^2+^ transformation. Data are expressed as mean ± SD of three independent experiments (*n* = 3). Figure S4: Fe^2+^ chelating activity of hydroalcoholic leaf extracts from *Salvia ceratophylloides* (Sc) and *Salvia officinalis* (So) plants grown in good-quality soil (Ctr) or in the same soil contaminated with two cadmium concentrations (Cd100 and Cd200). Data is expressed as the mean ± SD of three independent experiments (*n* = 3).
