# Supplementary material for: Differential Cadmium Responses in Two Salvia Species: Implications for Tolerance and Ecotoxicity
Source: Plants (Basel). 2026 Jan 25;15(3):375. doi: 10.3390/plants15030375 (PMC12899763; doi:10.3390/plants15030375)
Supplement: Supplementary file 1 [file plants-15-00375-s001.zip › plants-4087129-supplementary.pdf]

Supplementary Table S1: Analytical lines length (nm) used for analysis.

Element	nm	Element	nm	Element	nm
Cd	228,802	K	766.490	P	213,617
Cu	327.393	Mg	285.592	Zn	213.857
Fe	238.204	Mn	257.610	Ca	317.933

Supplementary Table S2: Operational conditions for ICP-OES analysis

Parameter	Conditions
Radiofrequency power (W)	1500
Plasma gas flow (L/min)	9
Auxiliary gas (L/min)	0.2
Nebulizer gas (L/min)	0.7
Sample uptake (mL/min)	1

Supplementary Table S3: Mean values ± SD (n=3) of macronutrients (K, P, Ca, Mg) and micronutrients (Fe, Zn, Mn, Cu) concentration measured in *Salvia ceratophylloides* (Sc) and *Salvia officinalis* (So) plants grown in good-quality soil (Ctr) or in the same soil contaminated with two concentrations of cadmium (Cd100 and Cd200). Different letters indicate significant differences between means ($p < 0.05$) based on two-way ANOVA followed by Holm-Sidak post-hoc test. Reported *p* values refer to the effects of species (Sp), cadmium concentration (Cd) and their interaction (Sp* Cd).* denotes interactions between factors.

	**K (mg g DW^{-1})**	**P (mg g DW^{-1})**	**Ca (mg g DW^{-1})**	**Mg (mg g DW^{-1})**	**Fe (µg g DW^{-1})**	**Zn (µg g DW^{-1})**	**Mn (µg g DW^{-1})**	**Cu (µg g DW^{-1})**
**Sc_Ctr**	79.7 ± 13.1	224.5 ± 32.8a	20.7 ± 6.2a	10.1 ± 2.5	8442.2 ±794.6a	2759.9 ± 305.7a	278.1 ± 44.2a	81.7 ± 16.1
**Sc_100Cd**	97.1 ±15.6	379.7 ± 60.2b	32.9 ± 9.1a	17.5 ± 4.5	6950.4 ± 665.5b	721.0 ± 105.2b	228.5 ± 37.1ab	87.5 ± 17.2
**Sc_200Cd**	69.3 ±11.1	394.1 ± 59.1b	32.2 ± 8.8a	18.7 ± 4.5	4533.5 ± 472.3c	796.4 ± 109.9b	174.8 ± 29.9b	82.1 ± 15.9
**So_Ctr**	123.0 ± 19.0	119.7 ± 22.6 c	111.9 ± 21.3b	36.5 ± 8.3	26321.5 ± 1942.2d	4104.4 ± 448.3c	403.3 ± 62.9c	83.0 ± 16.3
**So_100Cd**	187.7± 27.3	218.3 ± 37.4d	62.4 ± 13.1 c	36.7 ± 8.4	22449.7 ± 1888.1e	1706.5 ± 216.4d	342.1 ± 54.6c	79.7 ± 15.9
**So_200Cd**	155.1 ± 23.0	92.7 ± 18.3e	83.4 ± 16.8c	35.4 ± 8.0	417516.7 ±19573.3f	1417.6 ±186.2d	607.5 ±88.8d	95.1 ± 18.3
**Sp**	<0.01	<0.001	<0.001	<0.001	<0.001	<0.001	<0.001	0.810
**Cd**	0.071	<0.001	0.659	0.099	<0.001	<0.001	0.153	0.810
**Sp*Cd**	0.290	<0.001	0.01	0.08	<0.001	0.026	<0.001	0.590

Supplementary Table S4: Concentration of phenolic compounds in leaf extracts of *Salvia ceratophylloides* (Sc) and *Salvia officinalis* (So) grown in soil contaminated with two cadmium concentrations (Cd100 and Cd200). Data are reported as absolute concentration values and as percentage changes relative to the levels observed in plants grown in uncontaminated soil.

**Compound**	**Employed Standard for quantification**	**Sc (mg g^{-1} DW)**	**Sc_Cd100 (mg g^{-1} DW)**	**ΔSc_Cd100 (%)**	**Sc_Cd200 (mg g^{-1} DW)**	**ΔSc_Cd200 (%)**	**So (mg g^{-1} DW)**	**So_Cd100 (mg g^{-1} DW)**	**ΔSo_Cd100 (%)**	**So_Cd200 (mg g^{-1} DW)**	**ΔSo_Cd200 (%)**
Caffeic acid derivative	Caffeic acid	58.99	16.08	27.2	77.40	131.2	-	0	0	0.0	0
1-O-Caffeoyl glucose	Caffeic acid	4.29	3.14	73.2	2.84	66.2	8.49	7.48	88.1	7	82.4
Caffeic acid	Caffeic acid	11.02	6.30	57.2	12.30	111.6	19.08	17.51	91.8	21.04	110.3
Luteolin Rutinoside	Luteolin-7-O-glucoside	-	-	-	-	-	1.42	0.47	33.1	0.61	42.9
Lithospermic acid A	Caffeic acid	-	-	-	-	-	0.19	0.14	73.7	0.39	205.3
Quercetin 3-O-glucuronide	Quercitrin	-	-	-	-	-	0.75	0.95	126.7	1.01	134.7
Apigenin 7-O-Allosyl-(1->2)-glucoside	Apigenin	-	-	-	-	-	1.80	1.28	71.1	1,2	66.7
Luteolin-7-O-glucuronide	Luteolin-7-O-glucoside	16.79	5.22	31.1	3.76	22.5	53.40	42.78	80.1	43.76	81.9
Quercetin 3-O-glucuronide isomer	Quercitrin	-	-	-	-	-	0.63	0.19	30.2	0.34	53.9
Salvianolic acid C	Salvianolic acid B	-	-	-	-	-	12.46	9.1	73.0	9.4	75.4
Chrysoeriol-7-O-rutinoside	Luteolin-7-O-glucoside	-	-	-	-	-	5.99	4.01	66.9	3.18	53.1
Apigenin-O-pentoside	Apigenin	-	-	-	-	-	7.22	7.68	106.4	9.59	132.8
Rosmarinic acid	Caffeic acid	183.18	22.42	12.2	78.57	42.9	142.00	105.42	74.2	104.13	73.3
Salvianolic acid K	Salvianolic acid B	-	-	-	-	-	21.16	19.15	90.5	18.48	87.3

Supplementary Table S5: Percentage yields of *S. ceratophylloides* (Sc) and *S. officinalis* (So) leaf extracts. Plants were grown either in good-quality soil (Sc_Ctr and So_Ctr) or in soil contaminated with cadmium at two different concentrations (Sc_Cd100, Sc_Cd200 and So_Cd100, So_Cd200). For further details, see the main text.

	**Yield (w/w) %**
**Sc_Ctr**	42
**Sc_Cd100**	30
**Sc_Cd200**	31
**So_Ctr**	23
**So_Cd100**	27
**So_Cd200**	30

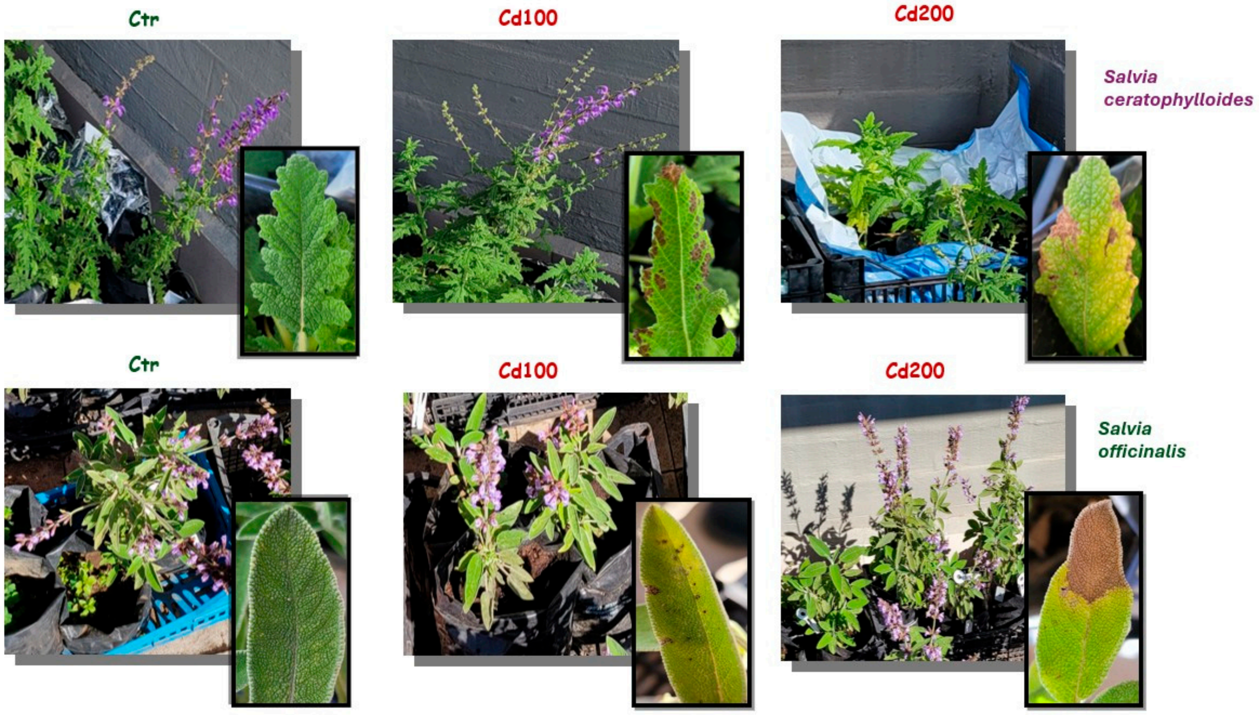

Supplementary Figure S1: Photographs of *Salvia ceratophylloides* and *Salvia officinalis* and details of their leaves recorded at the end of the experimental period. C: plants grown in uncontaminated soil. Cd100 and Cd200: plants grown under two cadmium contamination levels (for details, see Materials and Methods).

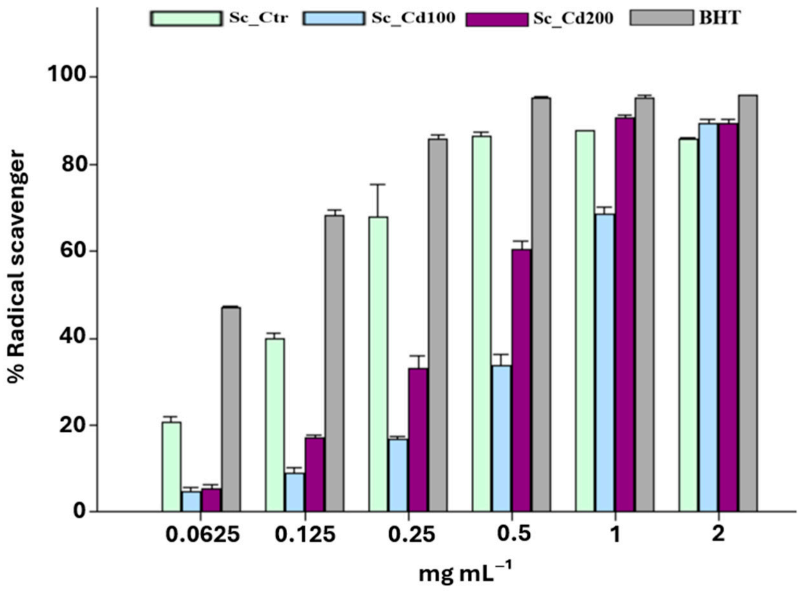

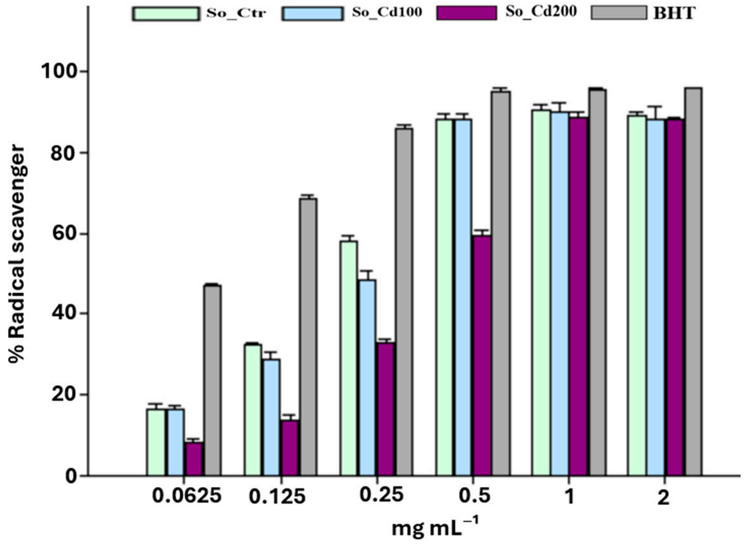

Supplementary Figure S2: Free radical scavenging activity (DPPH test) of hydroalcoholic leaf extracts from *Salvia ceratophylloides* (Sc) and *Salvia officinalis* (So) plants grown in good-quality soil (Ctr) or in the same soil contaminated with two concentrations of cadmium (Cd100 and Cd200). Data are expressed as the mean ± SD of three independent experiments (*n* = 3).

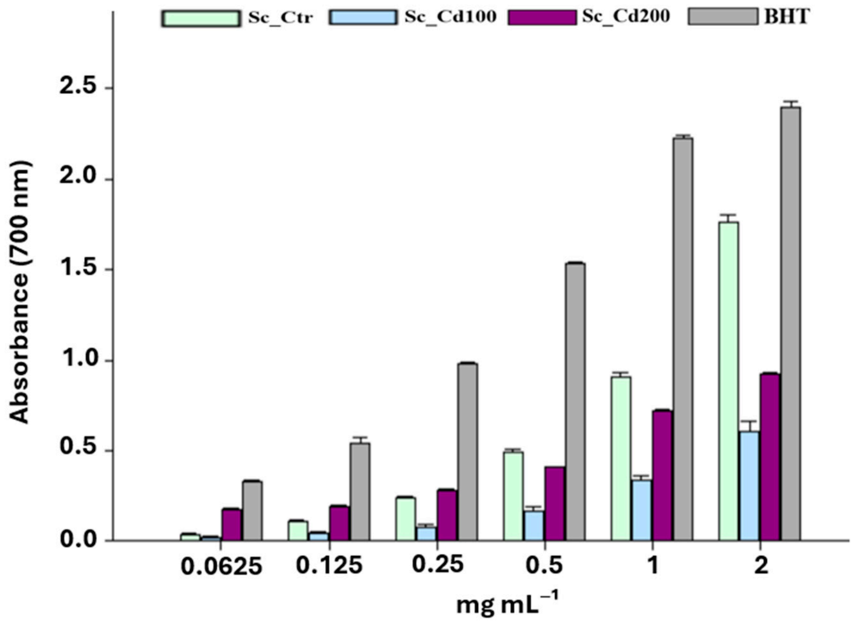

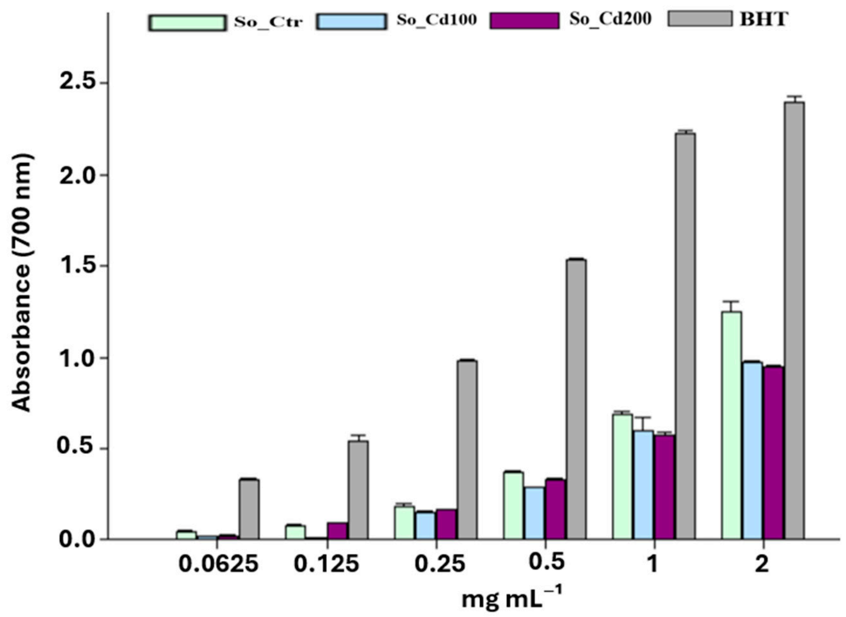

Supplementary Figure S3: Reducing power of hydroalcoholic leaf extracts from *Salvia ceratophylloides* (Sc) and *Salvia officinalis* (So) plants grown in good-quality soil (Ctr) or in the same soil contaminated with two cadmium concentrations (Cd100 and Cd200), evaluated by spectrophotometric detection of Fe^{3+}–Fe^{2+} transformation. Data are expressed as mean ± SD of three independent experiments (n = 3)

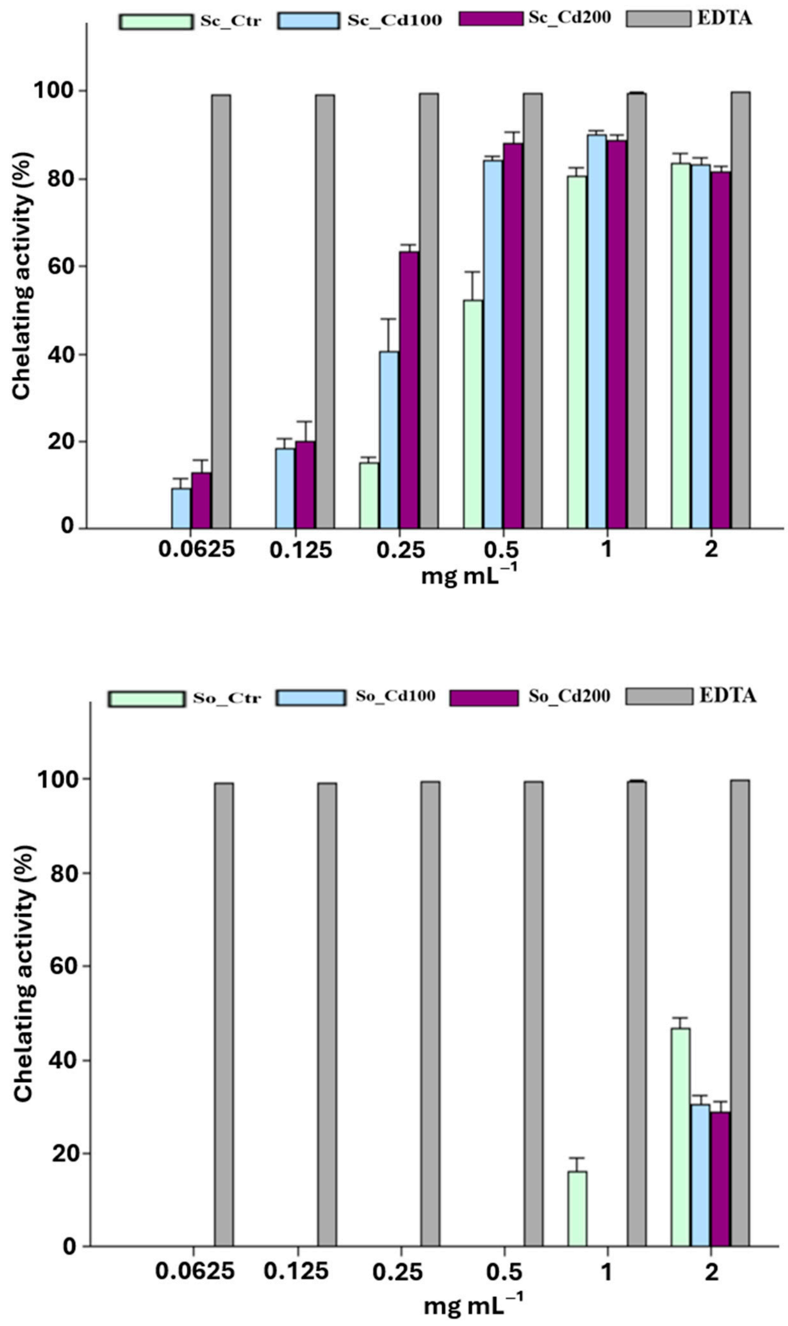

Supplementary Figure S4: Fe^{2+} chelating activity of hydroalcoholic leaf extracts from *Salvia ceratophylloides* (Sc) and *Salvia officinalis* (So) plants grown in good-quality soil (Ctr) or in the same soil contaminated with two cadmium concentrations (Cd100 and Cd200). Data is expressed as the mean ± SD of three independent experiments (*n* = 3).
